# PIAS1 Regulates Breast Tumorigenesis through Selective Epigenetic Gene Silencing

**DOI:** 10.1371/journal.pone.0089464

**Published:** 2014-02-24

**Authors:** Bin Liu, Samuel Tahk, Kathleen M. Yee, Randy Yang, Yonghui Yang, Ryan Mackie, Cary Hsu, Vasili Chernishof, Neil O'Brien, Yusheng Jin, Guoping Fan, Timothy F. Lane, Jianyu Rao, Dennis Slamon, Ke Shuai

**Affiliations:** 1 Division of Hematology-Oncology, Department of Medicine, University of California Los Angeles, Los Angeles, California, United States of America; 2 Department of Biological Chemistry, University of California Los Angeles, Los Angeles, California, United States of America; 3 Department of General Surgery, University of California Los Angeles, Los Angeles, California, United States of America; 4 Department of Pathology and Laboratory Medicine, University of California Los Angeles, Los Angeles, California, United States of America; 5 Department of Human Genetics, University of California Los Angeles, Los Angeles, California, United States of America; 6 Department of Obstetrics and Gynecology, University of California Los Angeles, Los Angeles, California, United States of America; Philipps University, Germany

## Abstract

Epigenetic gene silencing by histone modifications and DNA methylation is essential for cancer development. The molecular mechanism that promotes selective epigenetic changes during tumorigenesis is not understood. We report here that the PIAS1 SUMO ligase is involved in the progression of breast tumorigenesis. Elevated PIAS1 expression was observed in breast tumor samples. PIAS1 knockdown in breast cancer cells reduced the subpopulation of tumor-initiating cells, and inhibited breast tumor growth *in vivo*. PIAS1 acts by delineating histone modifications and DNA methylation to silence the expression of a subset of clinically relevant genes, including breast cancer DNA methylation signature genes such as cyclin D2 and estrogen receptor, and breast tumor suppressor WNT5A. Our studies identify a novel epigenetic mechanism that regulates breast tumorigenesis through selective gene silencing.

## Introduction

Both genetic and epigenetic alterations contribute to cancer development [1]–[3]. Tumor suppressors and epigenetic gatekeeper genes are frequently silenced by epigenetic mechanisms during tumor initiation and progression [3]–[5]. Extensive studies have been performed in the identification and characterization of altered DNA methylation in breast cancer development and progression. More than 100 genes have been reported to be aberrantly hypermethylated in breast tumors or breast cancer cell lines [1], [6]. Many of these genes play important roles in the regulation of cell cycle, apoptosis, angiogenesis, metastasis and tumor initiation. It has been proposed that breast cancer-specific DNA methylation signatures can extend our ability to classify breast cancer and predict outcome beyond what is currently possible [6]. Epigenetic therapy holds a promising potential for the successful treatment of cancer since epigenetic changes are reversible as opposed to mutations [7]. The approval of DNA methylation and histone deacetylase (HDAC) inhibitors for cancer treatment offers new promise for epigenetic therapy. However, these drugs are rather nonspecific, and the development of more effective strategies for epigenetic therapy requires a thorough understanding of the molecular specificity involved in epigenetic gene silencing.

Most tumors are composed of a mixture of cancer cells, and the heterogeneity of tumors is the major obstacle to effective cancer therapy. It has been demonstrated that a sub-population of cancer cells, referred to as tumor-initiating cells (TICs) or cancer stem cells, is tumorigenic when transplanted into immunosuppressed nonobese diabetic/severe combined immunodeficiency (NOD/SCID) mice [8]. TICs display some key properties of stem cells including self-renewal and multilineage differentiation [9]. In addition, TICs are found to be resistant to radiation and conventional chemotherapies [10]–[13]. Therefore, TICs may largely contribute to tumor cellular heterogeneity, tumor progression and tumor recurrence [14]–[17].

PIAS1 is a member of the PIAS (protein inhibitor of activated STAT) transcriptional regulator family that possesses SUMO (small ubiquitin-like modifier) E3 ligase activity [18]. Biochemical and genetic studies indicate that PIAS1 is a physiologically important transcriptional repressor of STAT1 and NF-kappaB [19]–[21]. PIAS1 is rapidly activated by phosphorylation on Ser90 residue in response to a variety of stimuli, including pro-inflammatory signals, TCR activation and growth factors. Activated PIAS1 is then recruited to gene promoters to repress transcription [22], [23]. Recent studies indicate that PIAS1 mediates a novel epigenetic regulatory mechanism to control natural regulatory T cell (Treg) differentiation. PIAS1 binds to the *Foxp3* promoter to maintain a repressive chromatin state through the recruitment of DNA methyltransferases (DNMTs) and HP1-gamma [24]. These findings indicate that this newly identified PIAS1 epigenetic mechanism plays an important role in T cell differentiation.

In this paper, we report that PIAS1 is important for breast tumorigenesis. Elevated PIAS1 expression was observed in breast cancer patient samples. PIAS1 knockdown in breast cancer cells inhibited tumor growth *in vivo*. Most interestingly, mechanistic studies indicate that PIAS1 suppresses a number of- genes clinically relevant to breast tumorigenesis through epigenetic mechanisms. These studies suggest that targeting the PIAS1 epigenetic signaling pathway may represent a novel therapeutic strategy for cancer treatment.

## Results

### Elevated PIAS1 expression in primary human breast cancer tissues

**Figure 1:**
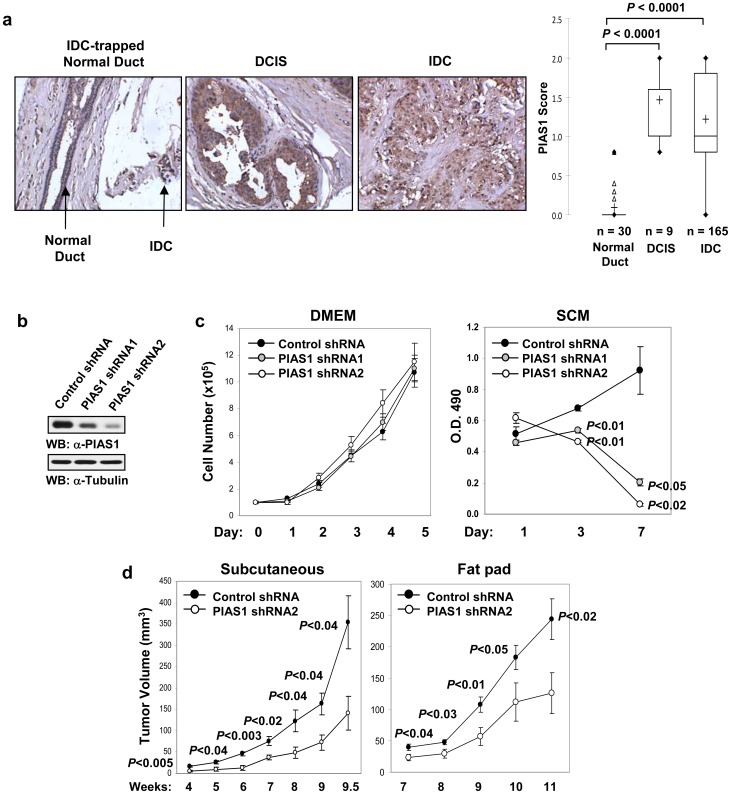
PIAS1 is important for tumorigenesis of breast cancer. (**a**) PIAS1 protein levels are increased in breast tumor samples as revealed by tissue microarray analysis. Left panel: Representative tissue microarray spot from morphologically normal duct, ductal carcinoma in situ (DCIS) and invasive ductal carcinoma (IDC). Right panel: a box and whisker plot of PIAS1 levels in various tissue samples. Total sample numbers (n) were indicated. *P* values are determined by non-parametric two-tailed Kruskal Wallis test with alpha level equals 0.05. “+”, the mean of each population; “Δ”, outliers. (**b**) Western blot analyses were performed with whole cell extracts from MDA-MB231 cells containing a control shRNA or two independent PIAS1 shRNAs (PIAS1 shRNA1 and 2). (**c**) The growth of MDA-MB231 control shRNA, PIAS1 shRNA1 and shRNA2 cells in DMEM supplemented with 10% FBS (left), or Stem Cell Media (SCM) (right) (mean ± SEM). Shown is a representative of 3 independent experiments. *P* values were determined by paired *t*-test. (**d**) *In vivo* tumorigenesis studies. MDA-MB231 cells containing a control shRNA or PIAS1 shRNA2 were injected into the female SCID-beige mice subcutaneously (left: 1×10^6^ cells/mouse; n = 4), or in fat pad (right: 2×10^5^ cells/mouse; n = 5). Shown is a representative of 3 independent experiments. Each data point represents mean ± SEM. *P* values were determined by non-paired *t*-test.

To test whether PIAS1 is involved in breast cancer progression, immunohistochemistry (IHC) tissue arrays were performed to examine the expression of PIAS1 protein in a panel of primary human breast tumor samples. PIAS1 is a nuclear protein, but it diffused to the cytoplasm under formalin fixation conditions (Figure S1 in File S1), a phenomenon observed with other nuclear proteins [25]. IHC analyses indicate that PIAS1 is significantly upregulated in primary breast cancer samples at early stages of breast ductal carcinoma *in situ* (DCIS) as well as invasive ductal carcinoma (IDC) (Fig. 1a).

### PIAS1 is important for breast tumorigenesis

To directly test whether PIAS1 plays a functional role in breast tumorigenesis, RNA interference approach was used to knockdown the expression of PIAS1 protein in MDA-MB231 cells. Stable cell lines expressing a scramble short hairpin RNA (control shRNA) or two independent PIAS1 shRNAs (shRNA1 and shRNA2) were obtained. Western blot analysis showed that PIAS1 expression was significantly suppressed by both PIAS1 shRNAs, although a more efficient inhibition by PIAS1 shRNA2 was observed (Fig. 1b). PIAS1 knockdown did not affect the growth of MDA-MB231 cells under the conventional serum-containing conditions (DMEM) (Fig. 1c, left panel). In contrast, when these cells were cultured under serum-free growth factor-enriched conditions (Stem Cell Media; SCM), which favor normal stem cells and more closely resemble primary tumors than the DMEM condition [26], PIAS1 knockdown significantly inhibited the survival of MDA-MB231 cells (Fig. 1c, right panel). To directly test the effect of PIAS1 knockdown on tumor growth in vivo, xenograft experiments were performed in SCID mice. PIAS1 knockdown significantly inhibited the tumor formation of MDA-MB231 cells in both the subcutaneous and the fat pad models (Fig. 1d), suggesting an important role of PIAS1 in the regulation of breast tumorigenesis.

### PIAS1 regulates the self-renewal of breast tumor initiating cells (TICs)

**Figure 2:**
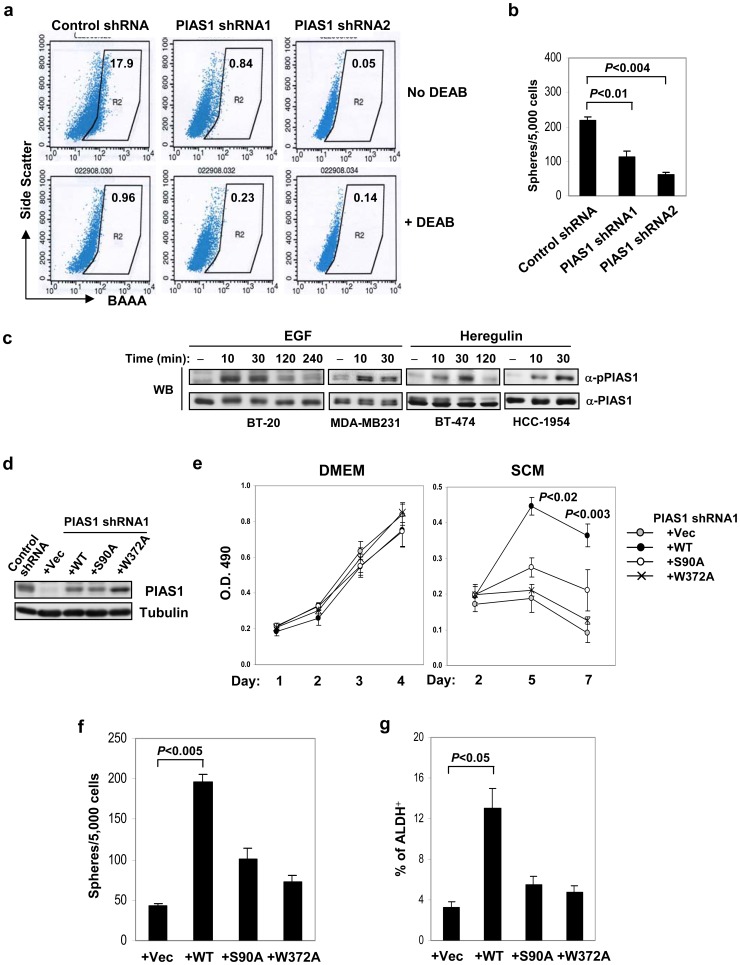
PIAS1 is important for the maintenance of the Tumor Initiating Cells (TICs) in MDA-MB231 cells. (**a**) Reduced ALDH^+^ population in PIAS1 knockdown cells using the ALDEFLUOR assay. Cells cultured in Stem Cell Media (SCM) for 25 days were incubated with ALDEFLUOR substrate (BAAA) with or without the specific inhibitor of ALDH, DEAB, to define the ALDH^+^ population (R2). The number indicates the percentage of the ALDH^+^ population. (**b**) Mammosphere assays. MDA-MB231 control shRNA and PIAS1 shRNA1 and shRNA2 cells were seeded in SCM on 35 mm petri dishes (5,000 cells/dish) and spheres were counted 7 days later. (**c**) PIAS1 is phosphorylated on Ser90 in response to EGF and Heregulin in breast cancer cells. Various breast cancer cells were starved for 16 h, then either untreated or treated with EGF (100 ng/ml) or Heregulin (15 ng/ml) for indicated time points. Western blot analyses were performed with whole cell extracts, using an antibody specific for Ser90-phosphorylated PIAS1 (anti-pPIAS1) or total PIAS1 (anti-PIAS1). (**d**) Reconstitution of MDA-MB231 PIAS1 shRNA1 cells with the lenti-viruses encoding the empty vector (Vec), wild type PIAS1 (WT), PIAS1 S90A mutant (S90A), or PIAS1 W372A mutant (W372A). Western blot was performed with whole cell extracts from these cells using anti-PIAS1 or anti-Tubulin. (**e**) The effect of WT or S90A and W372A PIAS1 mutants on cell proliferation and survival. MDA-MB231 cells as in **d** were seeded in DMEM supplemented with 10% FBS (left), or Stem Cell Media (SCM) (right) (mean ± SEM). Shown is a representative of 3 independent experiments. *P* values were determined by paired *t*-test. (**f**) Mammosphere assay. MDA-MB231 cells as in **d** were seeded in SCM at 5,000 cells/dish. Spheres were counted 7 days after plating. (**g**) ALDEFLUOR assay. MDA-MB231 cells as in **d** were cultured in SCM for 5 days, and the ALDH^+^ population was determined by the ALDEFLUOR assays. Shown in each panel is a representative of 3 independent experiments. Error bars represent SEM. *P* values were determined by paired *t*-test.

The finding that PIAS1 knockdown affects breast cancer cell survival specifically under the conditions that favor stem cell growth suggests a possibility that PIAS1 may play a role in the regulation of breast cancer stem cells/tumor-initiating cells (TICs). Previous studies suggest that the ALDH^+^ subpopulation of breast cancer cells is highly enriched in breast TICs [27]. ALDEFLUOR assays revealed that PIAS1 knockdown almost completely eliminated the ALDH^+^ population (Fig. 2a), supporting the hypothesis that PIAS1 knockdown inhibits breast TICs. To further test whether PIAS1 regulates breast TICs, the control and PIAS1 knockdown MDA-MB231 cells were subjected to mammosphere assays [14], [28], [29]. PIAS1 knockdown significantly inhibited the formation of mammospheres (Fig. 2b), suggesting that PIAS1 regulates the self-renewal of breast TICs.

### PIAS1 Ser90 phosphorylation and SUMO ligase activity are required for PIAS1-mediated regulation of breast TICs

Previous studies indicate that PIAS1 is activated by Ser90 phosphorylation to bind to chromatin and repress transcription of target genes in response to pro-inflammatory stimuli [22], a process that is dependent on the SUMO ligase activity of PIAS1. We explored whether PIAS1 can also be activated by growth factor signals. Western blot analysis revealed that PIAS1 became phosphorylated on Ser90 in response to EGF or Heregulin in various breast cancer cell lines, including MDA-MB231, BT-20, BT-474 and HCC-1954 (Fig. 2c). To test the importance of PIAS1 Ser90 phosphorylation and PIAS1 SUMO ligase activity in the regulation of breast TICs, PIAS1 shRNA1 knockdown MDA-MB231 cells were rescued with either an empty vector (Vec), wild type PIAS1 (WT), PIAS1 Ser90 mutant (S90A), or PIAS1 SUMO ligase defective mutant (W372A) through an shRNA escape approach, in which silent mutations were introduced into PIAS1 expression constructs to escape the inhibitory effect of PIAS1 shRNA. Western blot analysis indicated that the expression of WT or mutant PIAS1 proteins in the rescued cell lines was comparable to that of the MDA-MB231 control cells (Fig. 2d).

Consistent with the previous results (Fig. 1c), the introduction of either WT or S90A and W372A PIAS1 mutants did not affect cell growth under the conventional DMEM conditions (Fig. 2e, left panel). In contrast, when these cells were cultured under SCM conditions, only WT, but not the vector (Vec) or W372A mutant PIAS1 reconstituted cells, rescued cells from cell death (Fig. 2e, right panel). PIAS1 S90A mutant showed minor increase in cell survival, although the increase is not statistically significant (Fig. 2e, right panel). In addition, mammosphere assays were performed to examine the ability of WT or PIAS1 mutants to support the self-renewal of TICs. The introduction of PIAS1 WT into PIAS1 knockdown cells promoted the formation of mammospheres (Fig. 2f). The introduction of PIAS1 S90 or W372 mutant resulted in minor increases in mammospheres, although the increases are not statistically significant (Fig. 2f). Consistently, ALDEFLUOR assays indicated that PIAS1 WT, but not S90 or W372 mutant, restored the population of ALDH^+^ TICs (Fig. 2g). Taken together, these studies suggest that the observed inhibition of TICs in PIAS1 knockdown cells is due to the reduction of PIAS1 expression, and that both PIAS1 Ser90 phosphorylation and SUMO ligase activity are required for the maintenance of the breast TICs.

### PIAS1 selectively represses a subset of genes clinically relevant to breast cancer

We explored the molecular mechanism of PIAS1-mediated regulation of breast TICs. Gene profiling studies were performed to identify PIAS1 downstream genes involved in tumorigenesis. Total RNAs from the control and PIAS1 knockdown MDA-MB231 cells cultured under DMEM or SCM conditions were subjected to microarray analysis. Since PIAS1 is a transcriptional repressor and PIAS1 knockdown inhibited self-renewal of breast TICs under SCM conditions, we focused on the genes that were preferentially upregulated in PIAS1 knockdown cells under SCM conditions (Table S1 in File S1). Interestingly, among the group of genes strongly induced by PIAS1 knockdown, several genes are known to be clinically relevant to breast cancer, including breast cancer DNA methylation signature genes Cyclin D2 (*CCND2*) and Estrogen receptor (*ESR1*), candidate tumor suppressor *WNT5A*, progestagen-associated endometrial protein (*PAEP*), as well as leucine zipper, downregulated in cancer 1 (*LDOC1*). WNT5A, which signals through a non-canonical WNT pathway, is a candidate tumor suppressor in breast cancers [30]. The loss of WNT5A is associated with early relapse in invasive ductal breast carcinomas (IDC) and short recurrence-free survival. *PAEP* (also known as *GDA*/*PP14*) is an epithelial differentiation-related gene. *PAEP* expression is associated with a more favorable prognosis in breast and ovarian cancers, and PAEP inhibits breast tumor growth in SCID mice [31], [32]. *CCND2* is frequently silenced in a variety of human cancers, including breast and ovarian cancers, through promoter hypermethylation [33], [34]. *LDOC1* has been reported to be downregulated in pancreatic and gastric cancer cells [35].

**Figure 3:**
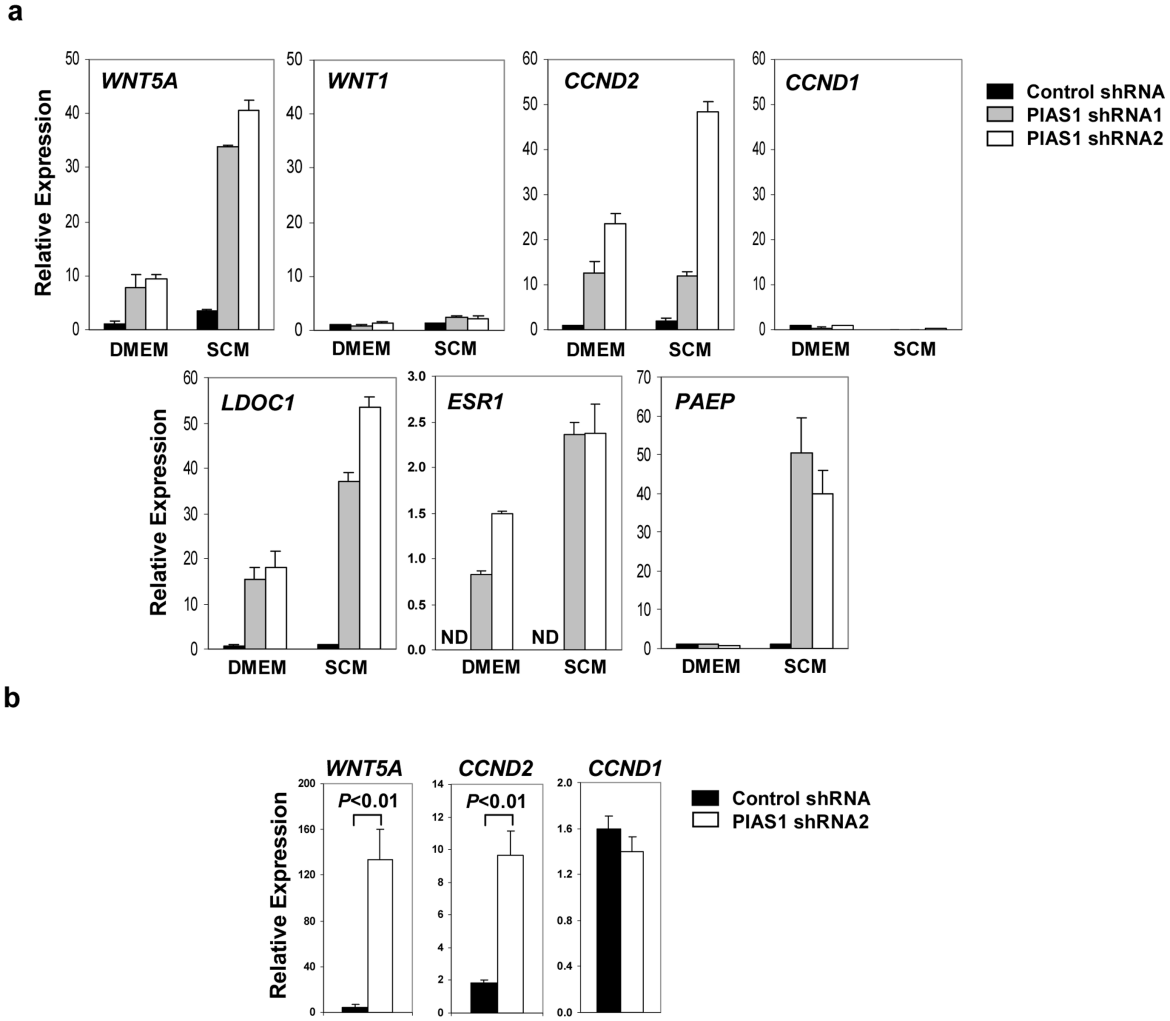
PIAS1 regulates the expression of a panel of tumor suppressor genes. (**a**) Real-time quantitative PCR (Q-PCR) assay. MDA-MB231 cells containing control shRNA, PIAS1 shRNA1 or shRNA2 were cultured in DMEM plus 10% FBS (DMEM) or Stem Cell Media (SCM) for 30 h, and total RNA was used for Q-PCR assays with gene-specific primers. The gene names are labeled at the top left of each panel. The data were normalized by beta-Actin (*ACTB*) and presented as “Relative Expression” as compared to that in control shRNA cells under DMEM condition, which was set as “1” except for the *ESR1* gene (the expression was not detectable in control shRNA cells). Shown is a representative of 3 independent experiments. Error bars represent SD. ND, not detected. See also Table S1 and Table S2 in File S1. (**b**) Same as in **a** except that total RNA from fat pad tumor xenograft samples were used (n = 5). Error bars represent SEM. *P* values were determined by non-paired *t*-test.

The induction of these genes identified by microarray was validated by quantitative real time PCR (Q-PCR) analysis in two independent PIAS1 knockdown MDA-MB231 cell lines (Fig. 3a and Table S2 in File S1). As a control, PIAS1 knockdown did not show significant effect on the expression of *WNT1* and *CCND1*, which show sequence homologies, but are functionally distinct in tumorigenesis, from *WNT5A* and *CCND2*, respectively. These results suggest that PIAS1 shows specificity in gene repression. Consistently, the transcription of *WNT5A* and *CCND2*, but not *CCND1*, was also elevated in PIAS1 knockdown xenograft tumor samples (Fig. 3b).

### PIAS1 promotes self-renewal of breast TICs through WNT5A suppression

**Figure 4:**
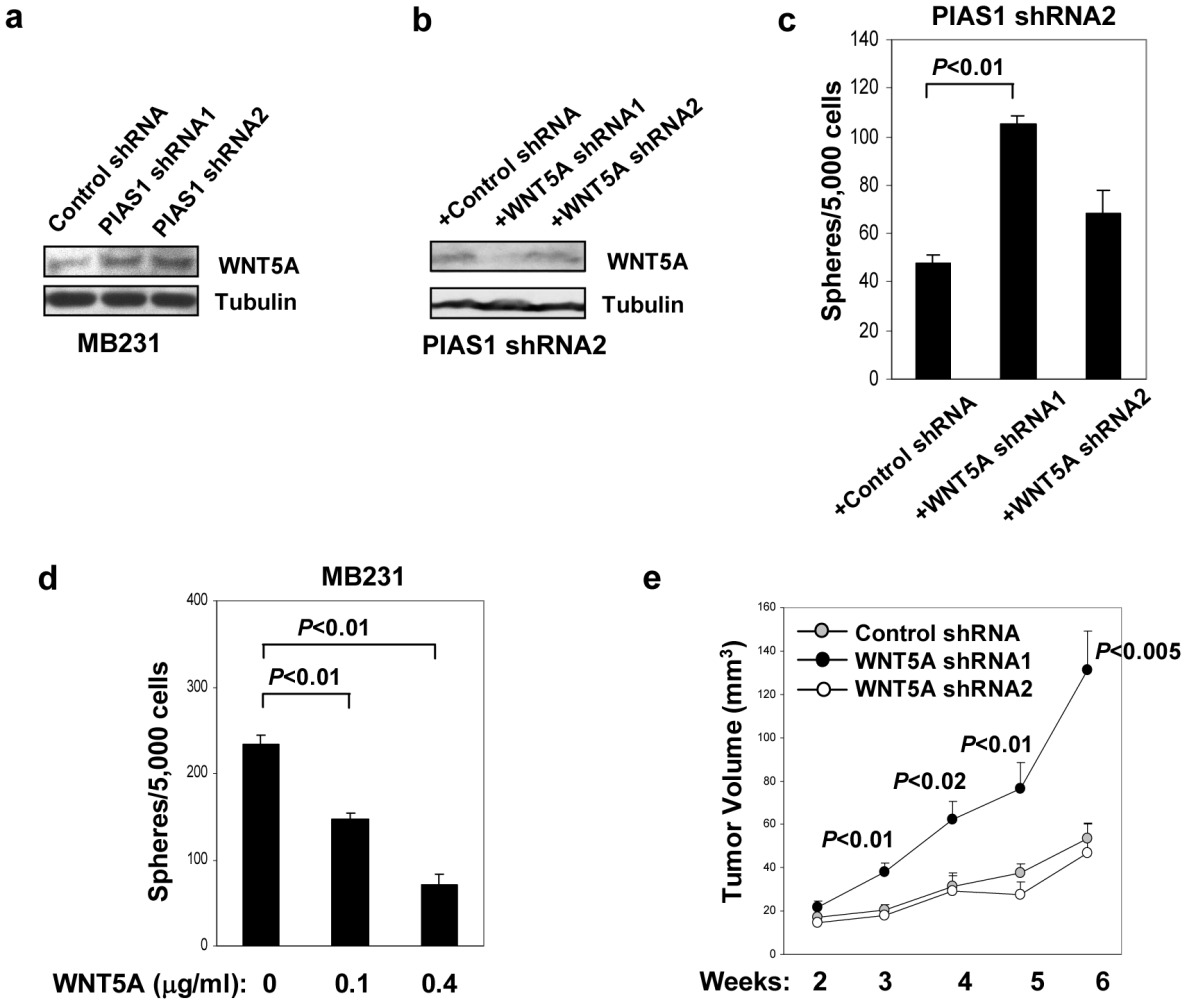
PIAS1-mediated *WNT5A* suppression is important for the maintenance of breast Tumor Initiating Cells (TICs). (**a**) Western blot analyses with whole cell extracts from MDA-MB231 cells containing control shRNA, PIAS1 shRNA1 or shRNA2 cultured in DMEM plus 10% FBS. (**b**) Western blot analyses with whole cell extracts from MDA-MB231 PIAS1 shRNA2 cells containing either a control shRNA, a WNT5A-specific shRNA (WNT5A shRNA1), or a non-working WNT5A shRNA (WNT5A shRNA2). (**c**) Mammosphere assay. Cells were seeded in Stem Cell Media (SCM) at 5,000 cells/dish, and spheres were counted 7 days later. Shown is a representative of 3 independent experiments. Error bars represent SEM. *P* values were determined by paired *t*-test. (**d**) Same as in **c** except that the parental MDA-MB231 cells were used with or without recombinant WNT5A treatment as indicated. (**e**) Tumorigenesis *in vivo*. Cells as in **b** were injected subcutaneously into SCID-beige mice (5×10^6^ cells/mice; n = 6). Shown is a representative of 3 independent experiments. Each data point represents mean ± SEM. *P* values were determined by non-paired *t*-test.

The WNT pathway is known to play a role in the regulation of self-renewal of stem cells [36]-[38]. Consistent with the gene expression results, higher levels of WNT5A protein were detected in PIAS1 knockdown MDA-MB231 cells (Fig. 4a). WNT5A shRNA was introduced into PIAS1 shRNA2 knockdown MDA-MB231 cells to inhibit *WNT5A* expression. While WNT5A shRNA1 efficiently inhibited the expression of *WNT5A*, WNT5A shRNA2 showed only a minor inhibition of *WNT5A* expression (Fig. 4b). Mammosphere assays showed that the suppression of *WNT5A* expression by WNT5A shRNA1 significantly enhanced the formation of mammospheres (Fig. 4c). Furthermore, the exogenous administration of recombinant WNT5A protein efficiently inhibited the mammosphere formation of parental MDA-MB231 cells (Fig. 4d). Consistently, the knockdown of WNT5A by shRNA1 significantly enhanced the tumor growth of PIAS1 knockdown MDA-MB231 cells *in vivo* (Fig. 4e). These studies support a role of WNT5A in PIAS1 knockdown-mediated inhibition of the self-renewal of breast TICs and breast tumorigenesis.

### PIAS1 promotes epigenetic gene silencing in breast cancer cells

**Figure 5:**
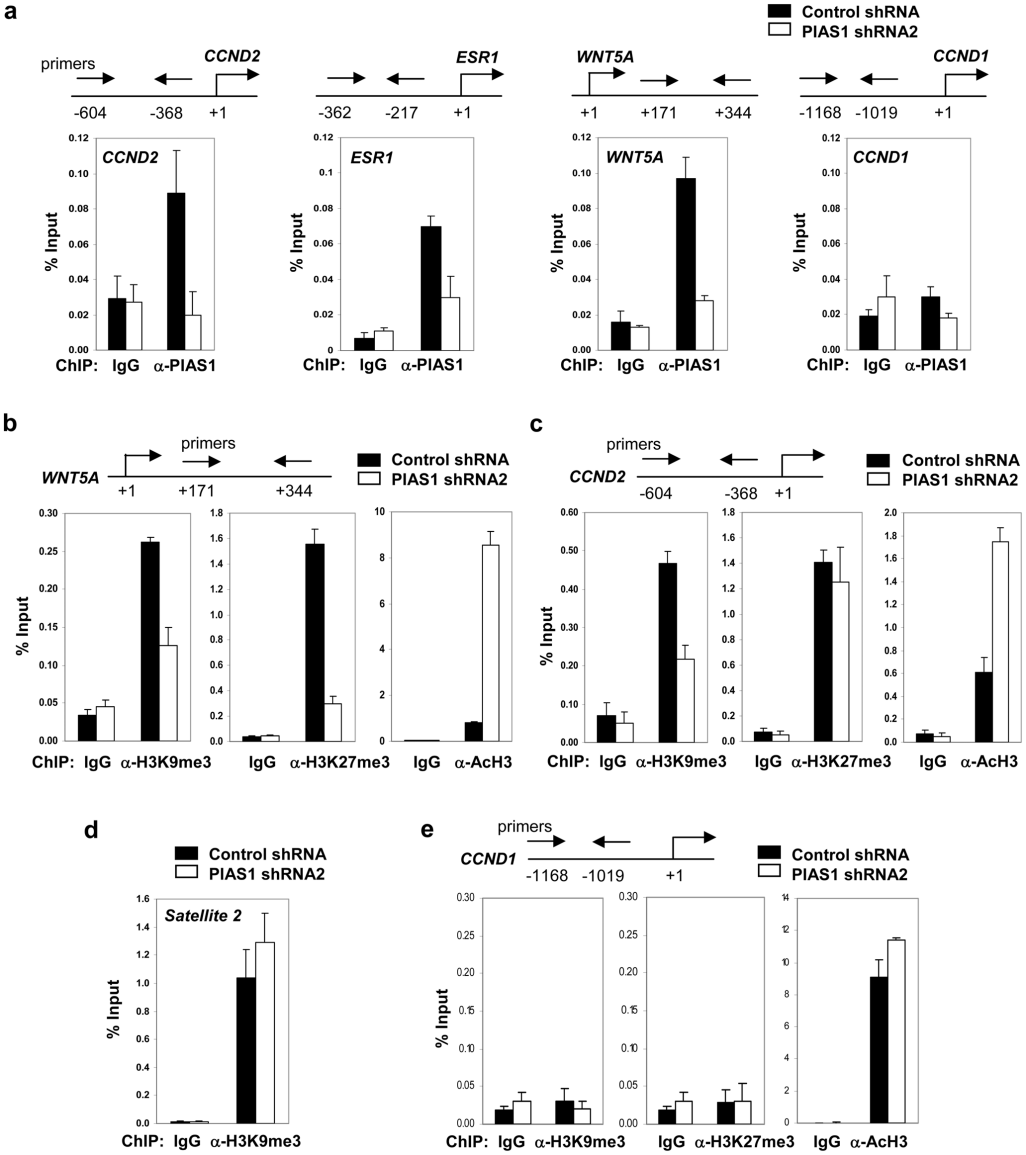
PIAS regulates the histone marks of the target genes. (**a**) Chromatin immunoprecipitation (ChIP) assay. Extracts from MDA-MB231 cells containing control shRNA, or PIAS1 shRNA2 were immunoprecipitated with anti-PIAS1 or IgG. The bound DNA was quantified by Q-PCR with gene-specific primers and presented as “percent of input” (% input). (**b**) Same as in **a** except that antibodies specific for acetylated histone H3 (AcH3), histone H3 trimethylated at Lys9 (H3K9me3), or histone H3 trimethylated at Lys27 (H3K27me3) were used, and the levels of these histone marks at the *WNT5A* loci were quantified. (**c**) Same as in **b** except that histone marks at the *CCND2* promoter were quantified. (**d**) Same as in **b** except that the level of H3K9me3 at the heterochromatin region *Satellite 2* was quantified. (**e**) Same as in **b** except that histone marks at the *CCND1* promoter were quantified. Shown in each panel is a representative of 3 independent experiments. Error bars represent SD.

Our recent studies showed that PIAS1 restricts nTreg differentiation by recruiting DNMTs to the *Foxp3* promoter to promote DNA methylation and epigenetic silencing [24]. We explored whether the PIAS1 epigenetic pathway also operates in breast cancer cells. Our previous results showed that PIAS1 inhibits the expression of *CCND2, ESR1* and *WNT5A*; but not *CCND1* (Fig. 3). Hypermethylation of the *CCND2, ESR1* and *WNT5A* loci has been reported in various cancer types [39]–[42]. Therefore, chromatin immunoprecipitation (ChIP) assays were performed to test whether PIAS1 was associated with the genomic loci with close proximity to the reported methylation regions of the *CCND2, ESR1* and *WNT5A* genes in MDA-MB231 cells. As shown in Fig. 5a, PIAS1 bound to the *CCND2, ESR1* and *WNT5A* loci in the control shRNA cells, while the binding was reduced in PIAS1 shRNA2 cells. Furthermore, PIAS1 was not associated with the *CCND1* promoter (Fig. 5a), consistent with the finding that PIAS1 does not affect the expression of *CCND1* (Fig. 3). Interestingly, PIAS1 knockdown resulted in a substantial increase of the active histone mark histone H3 acetylation (AcH3) on the *WNT5A* gene (Fig. 5b). In contrast, the repressive modifications, such as histone H3 K27 trimethylation (H3K27me3) and histone H3 K9 trimethylation (H3K9me3), were considerably reduced in PIAS1 knockdown cells (Fig. 5b). Similar changes in AcH3 and H3K9me3 were observed in the *CCND2* promoter (Fig. 5c). As a control, H3K9me3 was readily detectable in the centromeric satellite repeat, *Satellite 2*, a heterochromatin region [43] in MDA-MB231 cells (Fig. 5d). More importantly, the H3K9me3 level was not affected in PIAS1 knockdown cells, suggesting that PIAS1 does not affect global heterochromatin structure (Fig. 5d). In addition, while H3K9me3 and H3K27me3 were not detectable in the *CCND1* promoter, the AcH3 level was not affected by PIAS1 knockdown (Fig. 5e), consistent with the finding that PIAS1 does not affect the *CCND1* expression (Fig. 3). Taken together, these results suggest that PIAS1 regulates histone modifications of its target genes.

**Figure 6:**
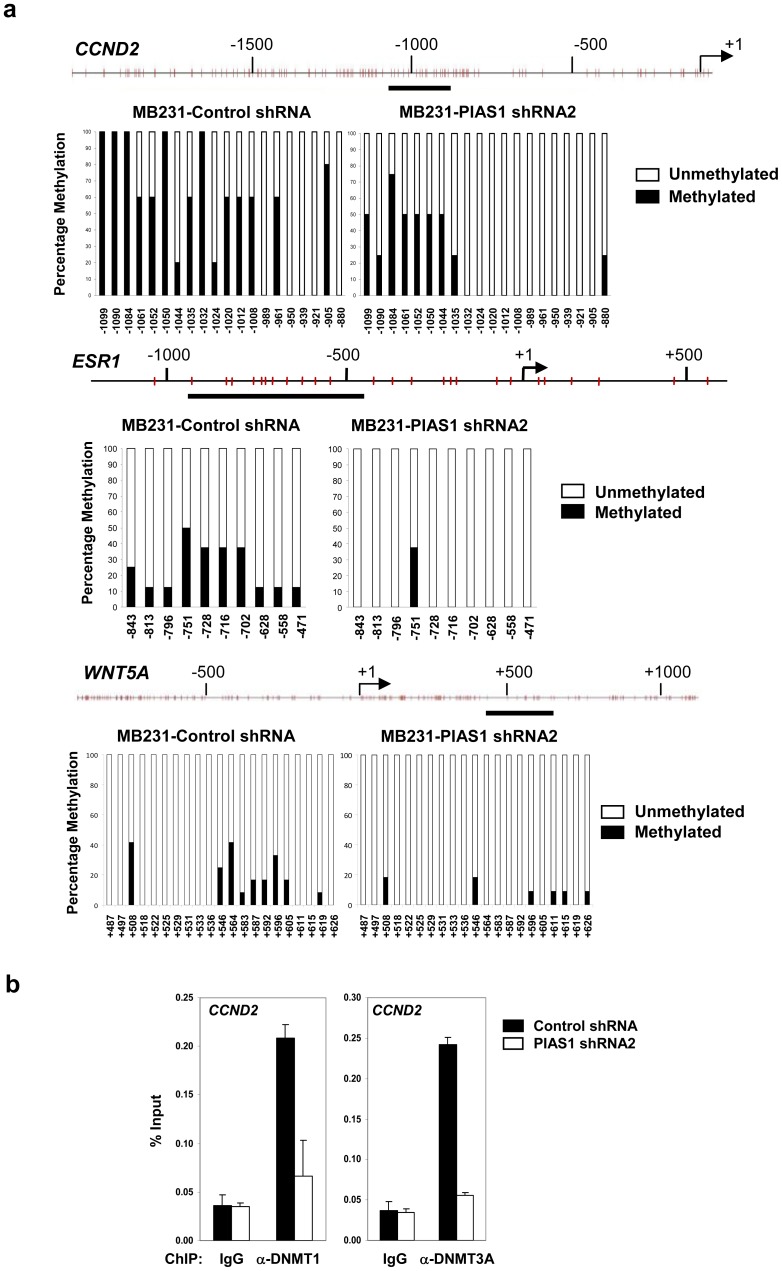
PIAS1 regulates DNA methylation status of the target genes. (**a**) DNA Methylation analyses of the indicated loci were performed by bisulfite conversion of genomic DNA from MDA-MB231 cells containing control shRNA or PIAS1 shRNA2. The ***x*** axis represents the positions of the CpG sites relative to the transcription start site (+1); the ***y*** axis represents the percentage. (**b**) Chromatin immunoprecipitation (ChIP) assay. Extracts from MDA-MB231 cells containing control shRNA, or PIAS1 shRNA2 were immunoprecipitated with anti-DNMT1, anti-DNMT3A or IgG. The bound DNA was quantified by Q-PCR with *CCND2* promoter-specific primers and presented as “percent of input” (% input). Shown in each panel is a representative of 3 independent experiments. Error bars represent SD.

*CCND2* and *ESR1* are signature genes that are frequently methylated in breast cancer [1]. Bisulfite sequencing analysis indicated that the promoter of *CCND2* was methylated in MDA-MB231 control cells, which was significantly reduced in PIAS1 knockdown cells (Fig. 6a). Similar reductions in DNA methylation were observed in *ESR1* and *WNT5A* genes (Fig. 6a). Consistently, ChIP assays indicated that both DNMT1 and DNMT3A bind to the *CCND2* promoter in the control MDA-MB231 cells, while the binding was compromised by PIAS1 knockdown (Fig. 6b). These studies suggest that PIAS1 recruits DNMTs to promote DNA methylation in breast cancer cells.

## Discussion

Although extensive studies have been performed in the identification and characterization of altered DNA methylation and epigenetic modifications in breast cancer development and progression [1], [4]–[6], the molecular mechanism involved in this process has not been understood. Studies described in this manuscript have identified a novel epigenetic control mechanism in promoting selective epigenetic silencing in breast cancer. Our results suggest that the PIAS1 epigenetic pathway, which has been previously shown to function in regulatory T cell differentiation [24], is up-regulated in breast cancer and is involved in promoting DNA methylation and epigenetic silencing of breast cancer signature genes such as *ESR1* and *CCND2*, as well as the breast tumor suppressor *WNT5A*.

Microarray analysis of PIAS1 knockdown breast cancer cells has uncovered an essential role of PIAS1 in the suppression of a group of genes previously known to be clinically relevant to breast cancer, such as *WNT5A* (Fig. 3). The WNT family of proteins can signal through the canonical beta-catenin-dependent or the non-canonical beta-catenin-independent pathway [36]–[38]. WNT5A belongs to the nontransforming class of the WNT gene family that activates non-canonical signaling pathways. The biology of WNT5A is cell-type dependent, and it has been reported that WNT5A may signal through different WNT receptors to cause different cellular responses [44]. Gene targeting studies indicate that WNT5A is required for normal mammary gland development, and *Wnt5a*-null ammary tissue shows an accelerated developmental capacity [45]. In addition, WNT5A overexpression inhibits tumorigenesis of uroepithelial cell carcinoma and suppresses mammary cell migration [46], [47]. The loss of WNT5A is associated with early relapse in invasive ductal breast carcinomas and short recurrence-free survival, supporting WNT5A as a candidate breast tumor suppressor [30]. The tumor suppressor function of WNT5A has also been suggested in other human cancers [48], [49]. In this report, we showed that PIAS1-mediated regulation of the self-renewal of breast TICs is largely achieved through the transcriptional repression of WNT5A. The exogenous administration of recombinant WNT5A protein to MDA-MB231 breast cancer cells suppressed mammosphere. Consistently, WNT5A inhibition by shRNA rescued PIAS1 knockdown-mediated suppression of mammosphere and tumor growth *in vivo* (Fig. 4). Our results suggest that the PIAS1-WNT5A pathway regulates the self-renewal of breast TICs.

PIAS1 is a nuclear SUMO E3 ligase that functions as a transcriptional repressor. PIAS1 is activated by Ser90 phosphorylation in response to proinflammatory stimuli. Activated PIAS1 is then recruited to gene promoters to repress transcription [22], [50]. In this report, we showed that PIAS1 is also phosphorylated/activated in response to growth stimuli, and the ability of PIAS1 to regulate the self-renewal of breast TICs requires PIAS1 Ser90 phosphorylation as well as PIAS1 SUMO E3 ligase activity (Fig. 2). Our studies suggest that PIAS1 may act as a sensor protein in the nucleus that responds to growth and inflammatory stimuli in the tumor microenvironment to regulate the self-renewal of TICs through epigenetic gene regulation.

**Figure 7:**
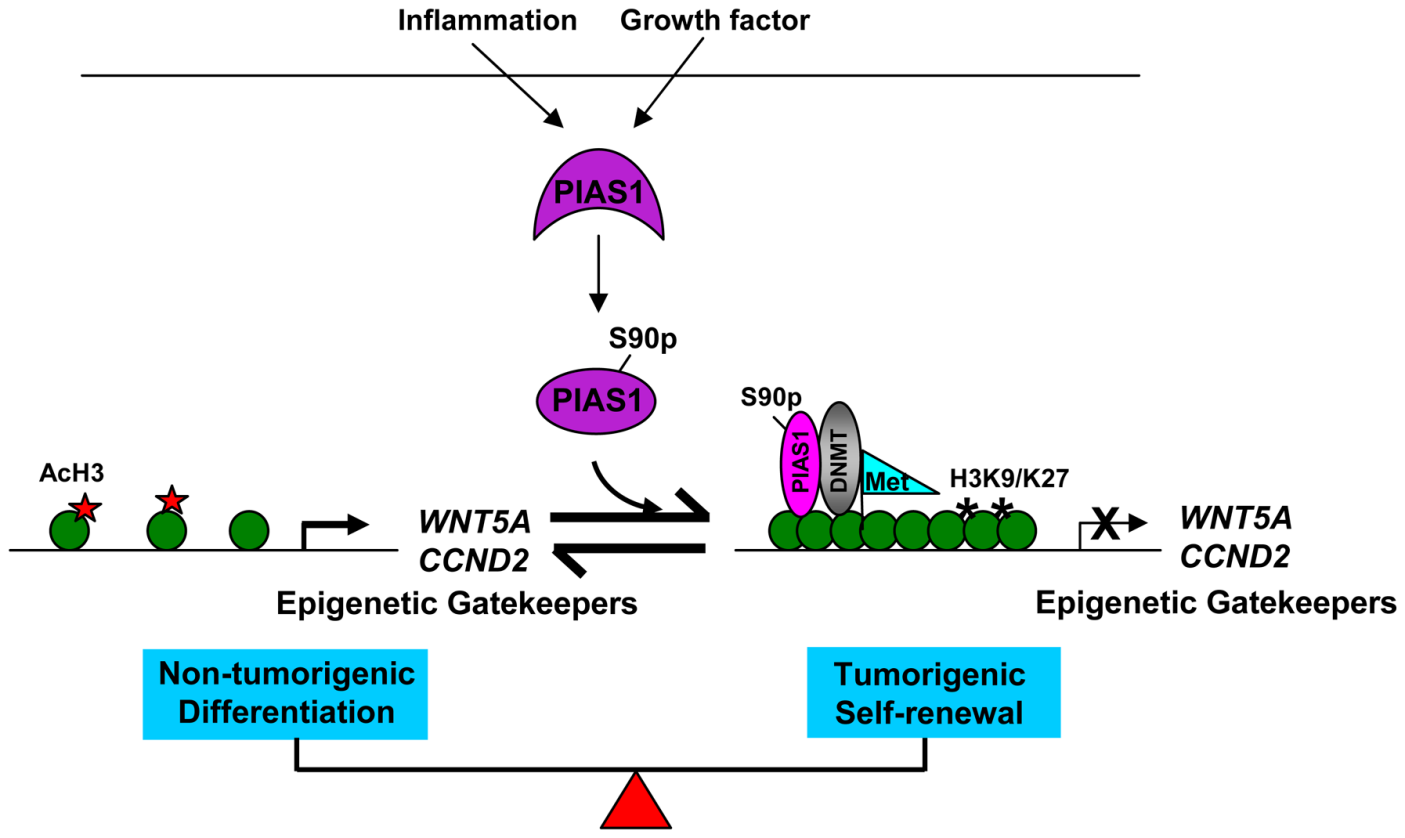
A proposed model of the function of PIAS1 in breast cancer. In response to growth factor and inflammatory signals, PIAS1 is activated via Ser90 phosphorylation (S90p), and recruited to the target gene promoters. PIAS1 represses the expression of epigenetic gatekeeper genes, such as *ESR1*, *WNT5A* and *CCND2*, by promoting inhibitory histone H3 lysine 9 and lysine 27 trimethylation (H3K9/K27) and DNA methylation (Met), while inhibiting acetylated histone H3 (AcH3). Therefore, PIAS1 promotes tumorigenesis by selective epigenetic gene silencing.

In conclusion, studies described in this paper suggest that PIAS1 plays an important role in promoting selective epigenetic silencing during breast tumorigenesis. It is possible that the PIAS1 epigenetic pathway may provide a link between inflammation and the development of breast cancer (Fig. 7). Targeting the PIAS1 epigenetic pathway may represent a novel therapeutic strategy for the treatment of breast cancer.

## Materials and Methods

### Mice, cell Lines and reagents

Tissue samples from breast cancer patients used for IHC analysis were purchased from commercial companies (Imgenex and Full Moon Biosystems). The work is exempt from Human Research since the data were analyzed anonymously. Human breast cancer cell lines MDA-MB231, BT-20, BT-474 and HCC-1954 were obtained from ATCC. MDA-MB231 cells were maintained in DMEM supplemented with 10% fetal bovine serum (FBS) and 1% Penicillin/Streptomycin. All other cells were maintained in RPMI supplemented with 10% FBS and 1% Penicillin/Streptomycin. Stem Cell Media (SCM) is composed of DMEM/F-12 (Cellgro) supplemented with 0.4% BSA, 1% Penicillin/Streptomycin, 2 mM Glutamine, 25 ng/ml human EGF (R&D), 25 ng/ml human basic FGF (R&D) and 5 ug/ml human insulin (Sigma). The following agents have also been used: Heregulin (Upstate), anti-pPIAS1 (Ser90-phosphorylated PIAS1) [22], polyclonal anti-PIAS1 [20], [51]; anti-Tubulin (Sigma), anti-WNT5A/B (Cell Signaling) and recombinant murine WNT5A protein (R&D). This study was carried out in strict accordance with the recommendations in the Guide for the Care and Use of Laboratory Animals of the National Institutes of Health. The protocol was approved by The UCLA Institutional Animal Care and Use Committee (Protocol Number: 1999-015-43A).

### shRNA knockdown and reconstitution

Oligonucleotides encoding a control small hairpin RNA (shRNA) or various shRNAs targeting PIAS1 or WNT5A were cloned under the control of the U6 promoter in the Lentiviral vector CS-CP for PIAS1 (containing a puromycin-resistant marker) or CS-CH for WNT5A (containing a hygromycin-resistant marker), which was modified from the CS-CG Lentiviral vector [52]. Lentiviruses were generated by co-transfecting 293T cells with shRNA constructs together with helper plasmids pCMV-VSV-G and pHR'8.9ΔVPR using the calcium phosphate method. The viral supernatant was collected 72 h post transfection, and used to infect various cancer cells. Cells were subjected to drug selection (puromycin: 2.5 ug/ml; hygromycin: 250 ug/ml) 48 h post infection. The target sequences of the shRNAs are: Control shRNA: GCACTACTGTCGATGACGA; PIAS1 shRNA1: GTTTCTGATAAACAAAACC; PIAS1 shRNA2: GAAACTATTCCATGGCAGT; WNT5A shRNA1: AGTGCAATGTCTTCCAAGT; WNT5A shRNA2: TATTAAGCCCAGGAGTTGC.

The wild type (WT), S90A and W372A mutant PIAS1 escape expression constructs were generated by insertion of WT or mutant PIAS1 cDNA fragments into the Lentiviral expression vector bearing a Hygromycin-resistant marker. These PIAS1 cDNAs carried 4 silent mutations that can escape the inhibition by shRNA without changing the codons of the protein (only the third nucleotide of each codon was altered). Lentiviruses were obtained as described above and target cells were infected with viral supernatant followed by Hygromycin selection.

#### *In vitro* mammosphere formation

*In vitro* mammosphere assays with MDA-MB231 cells were performed as described [12], [27]. Briefly, cells were seeded under SCM conditions at indicated densities on 35 mm petri dish. Fresh human EGF (25 ng/ml), basic FGF (25 ng/ml) and Insulin (5 ug/ml) were supplemented every 2 days. Spheres were counted under a microscope after 5–7 days of culture.

### Cell proliferation assay

For MDA-MB231 cell proliferation under DMEM conditions, cells were seeded in DMEM plus 10% FBS and 1% Penicillin/Streptomycin at a density of 1×10^5^ cells per well in 6 well plate, and stained with trypan blue and counted for viable cells everyday for 5 days. Alternatively, cells were seeded at a density of 3,000 cells per well in 96 well tissue culture plate (08-772-3; Fisher), and cell growth was determined everyday for 4–5 days by CellTiter96 AQueous One Solution Cell Proliferation Assay as instructed by the manufacturer (Promega). For cell growth under SCM conditions, cells were seeded in SCM at a density of 3,000 cells per well in 96 well non-treated microplate (12-565-226; Fisher), and supplemented with fresh human EGF (25 ng/ml), basic FGF (25 ng/ml) and Insulin (5 ug/ml) every 2 days. Cell growth was determined at indicated time points by CellTiter96 AQueous One Solution Cell Proliferation Assay as instructed by the manufacturer (Promega). Triplicates were performed for each time point of the growth curve.

### ALDEFLUOR assay

The ALDH^+^ cell population was determined using an ALDEFLUOR assay kit as instructed (StemCell Technologies). Briefly, cells grown under SCM condition were incubated with the ALDH substrate BAAA in ALDEFLUOR assay buffer in the presence or absence of the specific ALDH inhibitor diethylaminobenzaldehyde (DEAB) at 37°C for 45 min, followed by flow cytometry. The ALDH^+^ population of each sample was determined using its own negative control (DEAB containing sample) as a reference.

### *In vivo* tumorigenesis

Exponentially growing cells were trypsinized and resuspended in serum free-DMEM or RPMI, mixed with equal volume of Matrigel (BD Biosciences), and injected into the fat pad, or subcutaneously into the flank of 6–10 week old SCID beige mice (UCLA Department of Radiation Oncology) in a volume of 150 ul per site. Tumors were measured weekly with a caliper and tumor volume was calculated as width x length x height x 0.526.

### Tissue microarray analysis

Tissue microarray slides were obtained from Imgenex and Full Moon Biosystems, and immunohistochemistry (IHC) staining was performed using polyclonal anti-PIAS1 [20] as instructed by the manufacturers. “Normal duct” was defined as normal breast tissues from healthy individuals as well as histologically normal tissues adjacent to tumors. Semiquantitative assessment of PIAS1 staining was performed using a 0–2 scale (0 = negative; 1 = weak staining; 2 = strong staining) based on the average intensity per epithelial cell, and PIAS1 score was defined as the product of PIAS1 staining scale and the percentage of PIAS1 positive staining. A total of 3 independent tissue arrays containing 30–100 samples each were performed and the data were pooled. Statistic analysis was performed using non-parametric two-tailed Kruskal Wallis test with alpha level equals 0.05 for all tests, since both “Normal duct” and “IDC” populations are not normally distributed.

### Microarray analysis

MDA-MB231 cells containing a control shRNA or PIAS1 shRNA2 were cultured in DMEM plus 10% FBS (DMEM) or SCM for 30 h. Total RNA was prepared and subjected to microarray analyses using the human genome U133A 2.0 array chip (Affymetrix) as described [19]. The microarray data is presented in Table S1 in File S1 and has been deposited to Gene Expression Omnibus (GEO) database (GSE44024).

### Quantitative real time PCR (Q-PCR)

Quantitative real time PCR (Q-PCR) analyses were performed with breast cancer cells or tumor xenograft samples as described previously [19]. Briefly, total RNA was prepared using RNA STAT60 (Tel-Test). First strand complementary DNA was produced by reverse transcription (RT) of 1 ug total RNA using iScript cDNA synthesis kit (Bio-Rad). Q-PCR was carried out using the CFX96 real-time PCR detection system (Bio-Rad) in a final volume of 25 ul containing Taq polymerase, 1xTaq buffer, 125 uM dNTP, SYBR Green I (Molecular Probes) and gene-specific primers. Amplification conditions were: 95°C (3 min), 40 cycles of 95°C (10 s) and 61°C (30 s). Q-PCR data were analyzed by CFX Manager 2.0 software (Bio-Rad), and normalized by beta-Actin (*ACTB*). The results were presented as “Relative Expression” as compared to that in the control shRNA cells, which was set as “1”. Primers are listed in Table S2 in File S1.

### Chromatin immunoprecipitation (ChIP) assay

ChIP assays were performed using the ChIP analysis kit as instructed (Upstate). Briefly, cells grown in DMEM plus 10% FBS were cross-linked and lysed. Chromatin was sheared by sonication (10 s at 30% of the maximum strength for a total of six times). Cell extracts were immunoprecipitated with indicated antibodies, or IgG as a negative control. The binding of these factors to various DNA regions was quantified by quantitative real time PCR (Q-PCR) using the immunoprecipitates as templates and specific primers (Table S2 in File S1). The results were presented as “percent of input”. The following antibodies were used in the ChIP assay: normal rabbit IgG (sc-2027; Santa Cruz), anti-PIAS1 [51], anti-histone H3 trimethylated at Lys9 (H3K9me3) (17-625; Millipore); anti-histone H3 trimethylated at Lys27 (H3K27me3) (17-622; Millipore); anti-Acetylated histone H3 (AcH3) (17-615; Millipore); anti-DNMT1 (Ab13537; Abcam) and anti-DNMT3A (R0015-2; Abiocode).

### Bisulfite treatment and methylation analysis

Bisulfite modification of DNA was performed using EZ Methylation-Gold kit (Zymo Research). Bisulfite genomic sequencing (BGS) was conducted using cells grown in DMEM plus 10% FBS as described [39]–[42]. Taq DNA polymerase (Zymo Research) was used for PCR amplification using specific primers (Table S2 in File S1). The PCR conditions were as follows: 1 cycle of 95°C for 10 min, then 40 cycles of 95°C for 45 s, 56°C for 1 min, and 72°C for 1 min; and 1 cycle of 72°C for 10 min. Amplified products were cloned into pCR4-Topo (Invitrogen), with 8 to 12 colonies randomly chosen and sequenced.

## Supporting Information

File S1 Contains the following files: Figure S1, Table S1 and Table S2. **Figure S1.** Validation of the polyclonal anti-PIAS1 antibody by immunofluorescence. MDA-MB231 cells containing control shRNA or PIAS1 shRNA2 were fixed by 3 different methods as indicated, followed by staining with polyclonal anti-PIAS1. FMA, formaldehyde. **Table S1.** Microarray analysis. Fold induction is defined as the ratio of the expression levels of a given gene in PIAS1 shRNA2 vs. control shRNA cells. Genes with greater than 10-fold induction under Stem Cell Media (SCM) condition are shown. **Table S2.** Primers used for Q-PCR, ChIP and methylation.
(DOC)
