# Supplementary material for: PIAS1 Regulates Breast Tumorigenesis through Selective Epigenetic Gene Silencing
Source: PLoS One. 2014 Feb 24;9(2):e89464. doi: 10.1371/journal.pone.0089464 (PMC3933565; doi:10.1371/journal.pone.0089464)
Supplement: File S1 — Contains the following files: Figure S1, Table S1 and Table S2. Figure S1. Validation of the polyclonal anti-PIAS1 antibody by immunofluorescence. MDA-MB231 cells containing control shRNA or PIAS1 shRNA2 were fixed by 3 different methods as indicated, followed by staining with polyclonal anti-PIAS1. FMA, formaldehyde. Table S1. Microarray analysis. Fold induction is defined as the ratio of the expression levels of a given gene in PIAS1 shRNA2 vs. control shRNA cells. Genes with greater than 10-fold induction under Stem Cell Media (SCM) condition are shown. Table S2. Primers used for Q-PCR, ChIP and methylation. (DOC) [file pone.0089464.s001.doc]

## Supporting Information

## PIAS1 regulates breast tumorigenesis through

## selective epigenetic gene silencing

Bin Liu, Samuel Tahk, Kathleen M. Yee, Randy Yang, Yonghui Yang**, Ryan Mackie, Cary Hsu,** Vasili Chernishof, Neil O’Brien, Yusheng Jin, Guoping Fan, Timothy F. Lane, Jianyu Rao, **Dennis Slamon**, Ke Shuai

**Inventory of Supplementary Information:**

Figure S1 - related to Figure 1

Table S1 - related to Figure 3

Table S2 - related to Figure 3 and 5

**
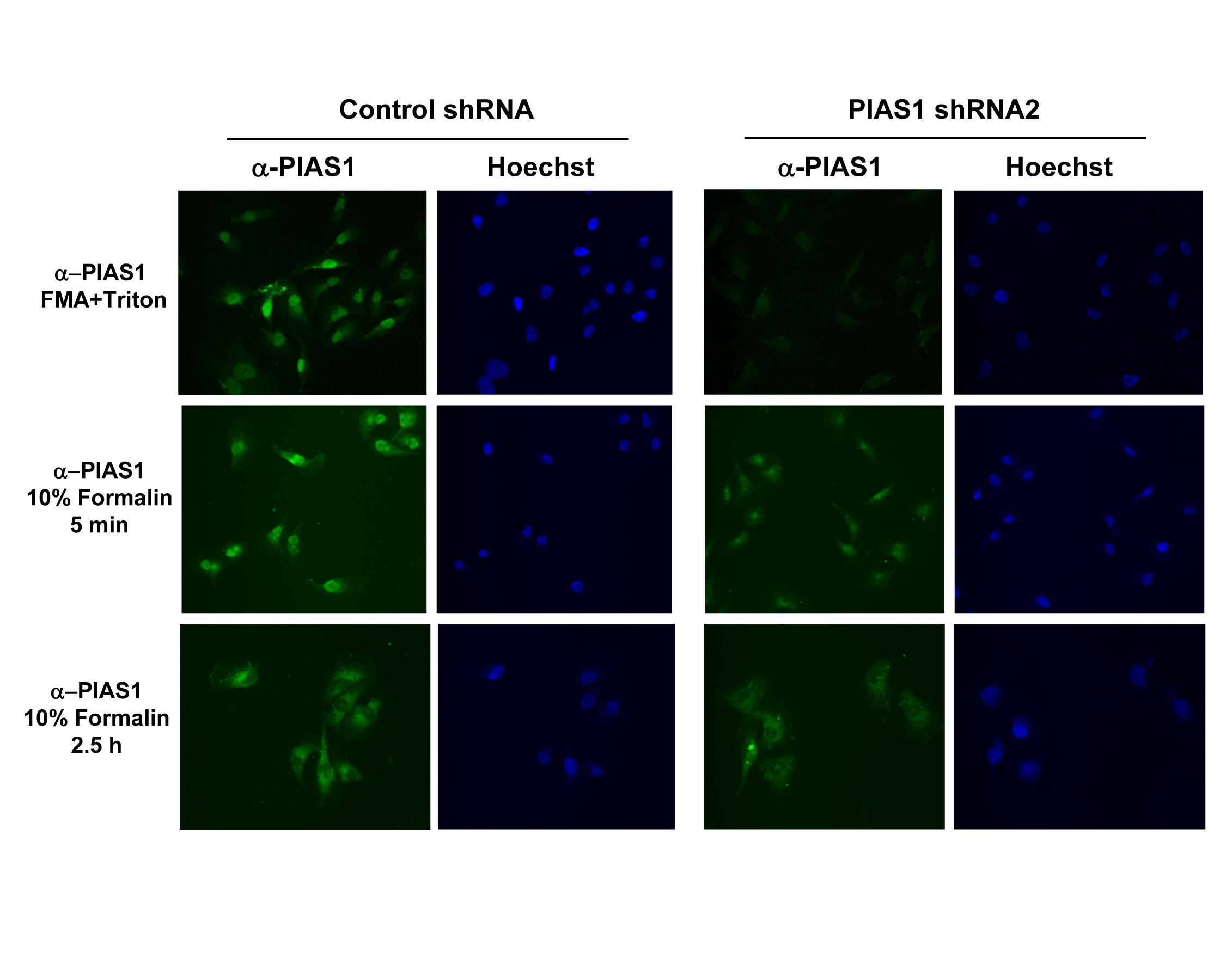
**

**Figure S1. Validation of the polyclonal anti-PIAS1 antibody by immunofluorescence.**

MDA-MB231 cells containing control shRNA or PIAS1 shRNA2 were fixed by 3 different methods as indicated, followed by staining with polyclonal anti-PIAS1. FMA, formaldehyde.

**Table S1. Microarray analysis**

Fold induction is defined as the ratio of the expression levels of a given gene in PIAS1 shRNA2 vs. control shRNA cells. Genes with greater than 10-fold induction under Stem Cell Media (SCM) condition are shown.

**Table S1.**  **Microarray analysis (continued)**

**Table S2.**  **Primers used for Q-PCR, ChIP and methylation.**

| **Species** | **Gene** | **5' or 3'** | **DNA sequence** |
| --- | --- | --- | --- |
| **Q-PCR primers:** |  |  |  |
| human | *WNT5A* | 5' | CAAGGGCTCCTACGAGAGTG |
|  |  | 3' | GCCAGCATGTCTTCAGGCTA |
|  | *WNT1* | 5' | CTCCTCCACGAACCTGCTTA |
|  |  | 3' | CGGATTTTGGCGTATCAGAC |
|  | *CCND2* | 5' | TCACCAACACAGACGTGGAT |
|  |  | 3' | ACGGTACTGCTGCAGGCTAT |
|  | *CCND1* | 5' | GCCATGAACTACCTGGACCG |
|  |  | 3' | TGATCTGTTTGTTCTCCTCCGC |
|  | *LDOC1* | 5' | GCTGCCAGGCTTACATCTTC |
|  |  | 3' | CCAGGTTGGTCCTGATCTC |
|  | *PAEP* | 5' | ACTATACGGTGGCGAACGAG |
|  |  | 3' | CAGGTACTGGCACATCATGC |
|  | *ESR1* | 5' | CGGCTCCGTAAATGCTACGA |
|  |  | 3' | AACATTCTCCCTCCTCTTCGG |
|  | *ACTB* | 5' | AGGCACCAGGGCGTGATGG |
|  |  | 3' | CATGGCTGGGGTGTTGAAGG |
| **ChIP primers:** |  |  |  |
| human | *WNT5A* | 5' | GGCCACAGTTGAGTAGTGGT |
|  |  | 3' | CAACTGTTCCACGGAGAGG |
|  | *CCND2* | 5' | TGTTCTGGTCCCTTTAATCG |
|  |  | 3' | AACGGATCCTAATCCTCCTG |
|  | *ESR1* | 5' | AAACCACCCATGTCCTATTTTG |
|  |  | 3' | AGGCTGGAAAGTACCCTATGCT |
|  | *CCND1* | 5' | TGGGGACCCTCTCATGTAAC |
|  |  | 3' | TAGAATTTGCCCTGGGACTG |
|  | *Satellite 2* | 5' | CATCGAATGGAAATGAAAGGAGTC |
|  |  | 3' | ACCATTGGATGATTGCAGTCAA |
| **Methylation primers:** |  |  |  |
| human | *WNT5A* | 5' | TGGGGTTGGAAAGTTTTAATTAT |
|  |  | 3' | ACTAAACACCTACCTTCATAAC |
|  | *CCND2* | 5' | TTTTGGAGTGAAATATATTAAAGGG |
|  |  | 3' | CCCCTACATCTAACAAACC |
|  | *ESR1* | 5' | TAATGTTTGGTAATAAAGTTTTTATTGG |
|  |  | 3' | AAAACCTTCTAAAATACATATAAATCAAAT |
